# The Multifaceted Inhibitory Effects of an Alkylquinolone on the Diatom *Phaeodactylum tricornutum*

**DOI:** 10.1002/cbic.201900612

**Published:** 2020-01-28

**Authors:** Lachlan Dow, Frederike Stock, Alexandra Peltekis, Dávid Szamosvári, Michaela Prothiwa, Adrien Lapointe, Thomas Böttcher, Benjamin Bailleul, Wim Vyverman, Peter G. Kroth, Bernard Lepetit

**Affiliations:** ^1^ Department of Biology University of Konstanz Universitätsstrasse 10 78467 Konstanz Germany; ^2^ Department of Biology Ghent University Krijgslaan 281/S8 9000 Ghent Belgium; ^3^ Institut de Biologie Physico-Chimique CNRS-Sorbonne Université 13 rue P. et M. Curie 75005 Paris France; ^4^ Department of Chemistry University of Konstanz Universitätsstrasse 10 78467 Konstanz Germany

**Keywords:** cytochromes, diatom–bacteria interactions, photosynthesis, quinolones, reaction mechanisms

## Abstract

The mechanisms underlying interactions between diatoms and bacteria are crucial to understand diatom behaviour and proliferation, and can result in far‐reaching ecological consequences. Recently, 2‐alkyl‐4‐quinolones have been isolated from marine bacteria, both of which (the bacterium and isolated chemical) inhibited growth of microalgae, suggesting these compounds could mediate diatom–bacteria interactions. The effects of several quinolones on three diatom species have been investigated. The growth of all three was inhibited, with half‐maximal inhibitory concentrations reaching the sub‐micromolar range. By using multiple techniques, dual inhibition mechanisms were uncovered for 2‐heptyl‐4‐quinolone (HHQ) in *Phaeodactylum tricornutum*. Firstly, photosynthetic electron transport was obstructed, primarily through inhibition of the cytochrome *b*_6_ *f* complex. Secondly, respiration was inhibited, leading to repression of ATP supply to plastids from mitochondria through organelle energy coupling. These data clearly show how HHQ could modulate diatom proliferation in marine environments.

## Graphical abstract

**Double trouble**: In the light, 2‐heptyl‐4‐quinolone (HHQ) primarily blocks photosynthetic electron flow at the cytochrome *b*_6_ *f* complex in the diatom *P. tricornutum*. In darkness, HHQ inhibits mitochondrial respiration, resulting in a decreased ATP supply, which ultimately leads to a decreased electric field strength in the thylakoid membranes inside the chloroplast of diatoms.

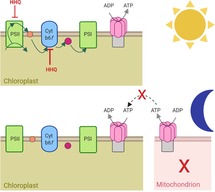

## Introduction

Diatoms are a class of unicellular algae found worldwide in aquatic environments, in which they are one of the chief primary producers.^1^ They persist in both benthic and pelagic habitats and are surrounded by a diverse array of other microbes, in particular, bacteria.^2^ Although a wide range of diatom–bacteria interactions have been identified, characterisation of the molecules and the corresponding modes of action that drive these interactions remains scarce. However, a class of bacterial secondary metabolites, 2‐alkyl‐4‐quinolones, some of which are used by bacteria as quorum sensing (QS) signals,^3^ have been identified as possessing algicidal effects on a variety of microalgae.^4^ Although not as ubiquitous as other QS compounds, such as *N*‐acyl homoserine lactones (AHLs), previous studies have reported that marine bacteria, particularly *Pseudoalteromonas* and *Alteromonas* species,^5^ but also freshwater and soil bacteria of the genera *Pseudomonas* and *Burkholderia*, produce quinolones.^6^

Quinolones reported most frequently from marine bacteria are 2‐pentyl‐4‐quinolone (PHQ)5c and the closely related 2‐heptyl‐4‐quinolone (HHQ).^4^, ^5^, ^7^ A recent study by Harvey et al. demonstrated that the marine bacterium *Pseudoalteromonas piscicida* was toxic to the microalga *Emiliania huxleyi*, due to the excretion of HHQ, with a half‐maximal growth inhibitory concentration (IC_50_) in the nanomolar range.5a In addition, two quinolones (2‐undecen‐1′‐yl‐4‐quinolone and 2‐undecyl‐4‐quinolone) were detected in extracts of another *Alteromonas* strain (KNS‐16), both of which inhibited the growth of a range of microalgae, with IC_50_ values varying between 1.6 and 200 μm, depending on the alga.^8^ Remarkably, the growth of algae in both of these studies was not only inhibited by living *P. piscicida* or *Alteromonas* KNS‐16 cells, but also if treated with the respective isolated quinolone. Furthermore, *Alteromonas* KNS‐16 was isolated directly from an algal bloom.^8^ This information suggests that 2‐alkyl‐4‐quinolones mediate bacteria–algae interactions through growth inhibition, and indeed may accumulate in the diffusion boundary that surrounds microalgae and associated bacteria, reaching locally high concentrations.2a, 9

Our understanding of the physiological effects of quinolones on diatoms remains scarce. In recent works treating diatoms with quinolones, it was shown that PHQ inhibited growth in *Cylindrotheca fusiformis*, *Thalassiosira weissflogii* and natural phytoplankton assemblages.5c The same compound was also found to inhibit growth and/or motility of the benthic diatoms *Amphora coffeaeformis*, *Navicula* sp. and *Auricula* sp.^10^ However, very few other alkylquinolones have been tested on diatoms, despite the diversity of alkylquinolones produced by marine bacteria. Furthermore, it is still not clear what causes quinolones to inhibit the growth of microalgae at all.

Nevertheless, the effects of alkylquinolones have been studied on other organisms previously.^11^ For instance, studies by Reil et al. found that synthetic quinolones, including 2‐alkyl‐4‐quinolones, were inhibitors of complex I (NADH:ubiquinone‐oxidoreductase; NADH: reduced nicotinamide adenine dinucleotide) and complex III (cytochrome *bc1* complex) in mitochondria.11b In addition, the same authors tested synthetic quinolones on isolated spinach thylakoids and reported that 2‐alkyl‐4‐quinolone *N*‐oxides were strong inhibitors of photosystem II (PSII), whereas 2‐alkyl‐4‐quinolones (such as HHQ or PHQ) were only weak inhibitors; this suggests that 2‐alkyl‐4‐quinolone *N*‐oxides are more potent inhibitors of photosynthesis than that of the corresponding 4(1*H*)‐quinolones in vascular plants.11c In addition, both compound groups were only weak inhibitors of the cytochrome *b*_6_ *f* complex.11c Furthermore, the alkylquinolones HHQ and PQS have been tested on a range of bacteria and yeasts upon which they had distinct effects on cell proliferation, motility and biofilm formation, and thus, indicating that their effects are species specific.11a

The observations that quinolones produced by marine bacteria can inhibit growth in certain microalgae has prompted us to investigate their effects on diatoms in detail. We aimed to study how structural analogues would affect diatom growth, and whether a mode of action could be observed with diatoms in vivo. Herein, we present work regarding a number of native bacterial quinolones, namely, 2‐heptyl‐4‐quinolone *N*‐oxide (HQNO), as well as the *Pseudomonas* QS signals HHQ and 2‐heptyl‐3‐hydroxy‐4‐quinolone (PQS), and the 2‐nonyl congeners 2‐nonyl‐4‐quinolone (NHQ) and 2‐nonyl‐4‐quinolone *N*‐oxide (NQNO). To account for the different environments in which quinolones have been detected, we selected three diatoms from different aquatic ecosystems for our initial screening: *Cylindrotheca closterium* is a marine biofilm‐forming diatom often found in the benthos of the intertidal zone. In contrast, *Phaeodactylum tricornutum* is a planktonic diatom, which was originally isolated in coastal water and is commonly used as a model organism.^12^ Additionally, *Achnanthidium minutissimum* represents a biofilm‐forming freshwater diatom. Because Reil et al. identified an inhibition of photosynthesis by certain quinolones in isolated spinach thylakoids, as well as an inhibition of respiration in isolated mitochondria of non‐photosynthetic organisms,11c we used a variety of experiments to probe not only photosynthesis, but also respiration. In doing so, we demonstrate in vivo how the reported growth impairment of diatoms by quinolones is achieved through a simultaneous specific inhibition of both photosynthesis and respiration.

## Results

### Inhibition of diatom growth by quinolones

**Figure 1:**
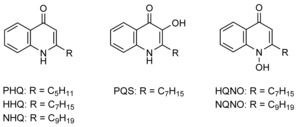
Structures of 2‐alkyl‐4‐quinolones discussed herein.

**Figure 2:**
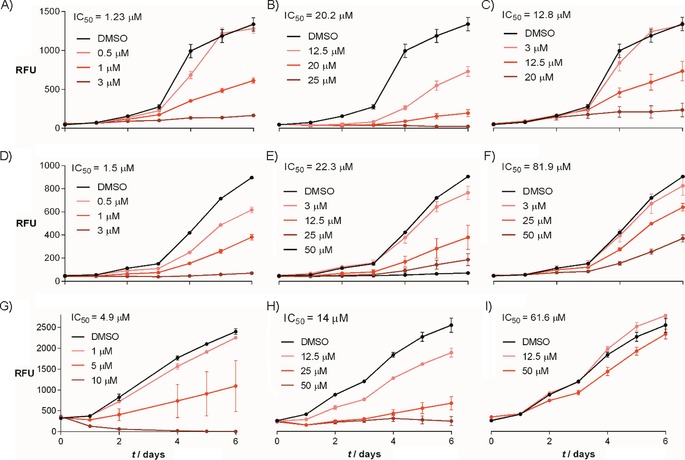
Growth curves of *C. closterium* (A–C), *A. minutissimum* (D–F) and *P. tricornutum* (G–I) exposed to a range of concentrations of HHQ (1st column), PQS (2nd column) and HQNO (3rd column) over 6 days, with day 0 depicting the day compounds were added to the culture. Tested concentrations varied between diatoms and compounds. Growth was followed by measuring chlorophyll fluorescence, defined as arbitrary relative fluorescence units (RFUs). Each data point represents the mean of three replicates with error bars showing the standard deviation. IC_50_ values are shown at the top of each graph.

Intrigued by the reported bioactivity of quinolones on microalgae, we tracked the growth of three diatoms treated with quinolones at a range of concentrations (0.125–100 μm). The quinolones used varied with regard to their N‐oxidation, 2‐alkyl chain length and 3‐hydroxyl group (Figure 1). Five quinolones, HHQ, NHQ, PQS, HQNO and NQNO, were applied to cultures of *P. tricornutum*, *C. closterium* and *A. minutissimum*. Of the three quinolones tested with a heptyl side chain (HHQ, PQS and HQNO), all diatoms were most sensitive towards HHQ, with IC_50_ values between 1 and 5 μm for each respective species (Figure 2 A, D, G). HHQ completely inhibited growth at concentrations as low as 3 μm in *C. closterium* (Figure 2 A) and *A. minutissimum* (Figure 2 D), and at 10 μm in *P. tricornutum* (Figure 2 G). Relative to HHQ, the IC_50_ values of PQS were found to be 3 to 16 times higher (Figure 2 B, E, H). *A. minutissimum* and *P. tricornutum* were less sensitive to the *N*‐oxide HQNO compared to that of PQS (Figure 2 F and I), whereas *C. closterium* was more sensitive (Figure 2 C).

Diatoms were more sensitive to both NHQ and NQNO (Figure S1 in the Supporting Information) compared with that of their hept‐2‐yl counterparts (HHQ and HQNO), which indicated that a longer alkyl side‐chain length increased the toxic effect of these compounds. This observation substantiates a quantitative structure–activity relationship study to test quinolones on spinach thylakoids, which found that quinolones reached their maximum photosynthetic inhibitory potential at an alkyl chain length of 11 carbon atoms.11c However, unlike experiments with spinach thylakoids, diatoms displayed decreased sensitivity towards *N*‐oxide quinolones: just as HHQ was more potent than that of its *N*‐oxide counterpart, so too was NHQ more potent than that of NQNO.

Taken together, these observations showed that structural features of quinolones influenced the respective growth response of diatoms; *N*‐oxide‐ or hydroxyl‐functionalised quinolones were less potent than that of non‐functionalised quinolones. This trend was consistent among all diatoms tested, although sensitivity varied between species, of which *C. closterium* was the most sensitive, followed by *A. minutissimum* and *P. tricornutum*.

### Blocking of electron flow between PSII and PSI by HHQ

**Figure 3:**
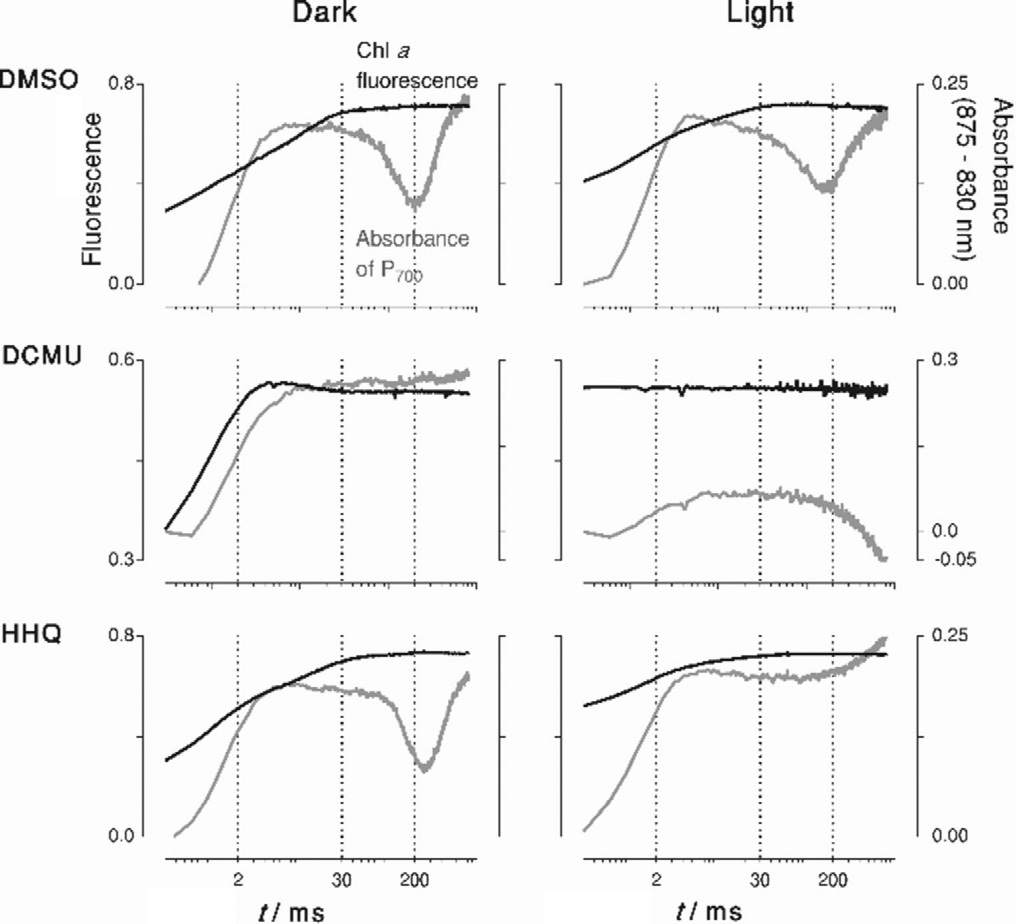
Simultaneously measured fast fluorescence kinetics of PSII (black) and absorbance of P_700_ (grey, indicative of P_700_ redox state) of *P. tricornutum*. Cells were treated with DMSO (control), 40 μm 3‐(3,4‐dichlorophenyl)‐1,1‐dimethylurea (DCMU; positive control) or 5 μm HHQ. The activities of PSI and PSII were measured through Dual‐PAM during a multi‐turnover pulse (800 ms) with dark‐adapted cells (dark, left column) and the same cells adapted to low actinic light (light, right column, 70 μmol photons m^−2^ s^−1^). Data from one representative sample of each treatment are shown. Data are plotted on a logarithmic *x* axis, with dotted lines indicating crucial time points of the fast fluorescence curve at 2, 30 and 200 ms. Graphs of the same treatments share the same *y*‐axis range, with the left *y* axis indicating PSII fluorescence and the right *y* axis indicating P_700_ absorbance.

The strong inhibitory effect of HHQ on all three diatom species, and its relevance towards other interactions between microalgae and bacteria, prompted us to investigate the mode of action of HHQ in *P. tricornutum* in detail. To this end, we first investigated the effect of HHQ on the activities of photosystems II and I (PSII and PSI, respectively) by measuring the fluorescence induction of PSII and absorbance changes of P_700_ (the special pair of chlorophyll in the reaction centre of PSI) simultaneously, using dual pulse amplitude modulated (Dual‐PAM) fluorometry. Fluorescence comes mainly from PSII and its intensity depends on the redox state of Q_A_—the primary quinone electron acceptor of PSII—which takes up electrons originating from the water‐splitting reaction at the oxygen‐evolving complex. Meanwhile, changes in absorbance (*λ*=875 nm minus that at *λ*=830 nm) provide information about the redox state of P_700_ within PSI.^13^ Using these parameters, the PSII and PSI kinetics were measured in dark‐adapted and low‐light‐adapted cells. HHQ was tested at a range of concentrations; as an example, Dual‐PAM data at 5 μm (the IC_50_ value for *P. tricornutum*) are shown in Figure 3 (the quantum yield, *Y*(II), from the other concentrations tested are shown in Figure S2). DMSO and DCMU (a potent PSII inhibitor), served as respective negative and positive controls (Figure 3). Typically, the change in absorbance of P_700_ (Figure 3, grey line) consists of three phases during the saturating pulse, in both dark‐ and light‐adapted control cells: a fast increase in absorbance in the first 30 ms, which is indicative of photo‐oxidation of PSI; followed by a partial decrease in absorbance between 30 and 200 ms, which indicates a reduction of PSI; again followed by an increase in absorbance, showing the re‐oxidation of PSI (>200 ms). In the presence of HHQ, the transient reduction of P_700_ between 30 and 200 ms was still present in dark‐adapted cells, but was suppressed in light‐adapted cells. This reduction transient has previously been assigned to electron flow from PSII in *P. tricornutum*,^14^ which is in agreement with its suppression in the presence of DCMU in our data (Figure 3). In this regard, the absence of a P_700_ reduction transient in light‐adapted cells treated with HHQ suggests that these molecules inhibit an electron‐transfer step between PSII and PSI. However, in the DCMU treatment, the transient reduction of PSI was also abolished in dark‐adapted cells, which suggested that HHQ had a different mode of action to DCMU. This observation was supported by the maximum quantum yield of PSII, which was not significantly affected in dark‐adapted cells by HHQ treatment (Figure S2), whereas DCMU treatment induced a clear decrease in PSII quantum yield.

**Figure 4:**
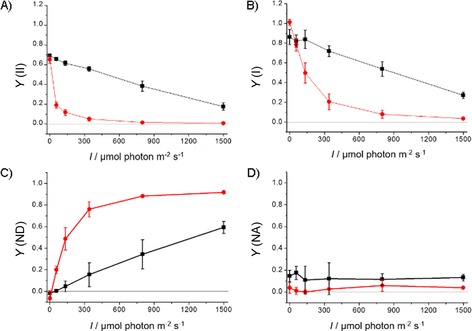
Light dependency of the quantum yields of A) PSII (*Y*(II)) and B) PSI (*Y*(I)), as well as C) PSI donor site limitation and D) PSI acceptor site limitation under steady‐state illumination in *P. tricornutum* (*I*=light irradiance). ▪: control samples (DMSO); •: HHQ treated (50 μm). Error bars represent standard deviation (*n*=3).

To confirm this, and to identify the molecular target of HHQ in the electron‐transfer chain of *P. tricornutum*, we used a Joliot‐type spectrometer (JTS) and saturating concentrations of HHQ (50 μm, see Figure S2). Firstly, we measured the light dependency of the quantum yields of PSII and PSI (*Y*(II) and *Y*(I), respectively), in the presence and absence of HHQ (Figure 4). Again, the maximal yields of PSI and PSII were not significantly inhibited by HHQ in the dark (Figure 4 A, B). However, at all light irradiances, the quantum yields of both photosystems were significantly lower in the presence of HHQ relative to that of the control (Figure 4 A, B). This translates into a lower electron‐transfer rate through both PSII and PSI in the presence of HHQ, regardless of the irradiance (Figure S3). Under the same conditions, we also measured the acceptor‐ and donor‐side limitations of PSI (see the Experimental Section). The data clearly show that the decrease of *Y*(I) is paralleled by an increase of the donor‐side limitation (*Y*(ND)), that is, P_700_ is more oxidised in HHQ‐treated samples (Figure 4 C). In addition, PSI is not limited by the acceptor side because the proportion of non‐photo‐oxidisable P_700_ (*Y*(NA)) is almost non‐existent, regardless of light irradiance (Figure 4 D). These data rule out the possibility that HHQ inhibits the PSI acceptor site or beyond (e.g., the ferredoxin NADP reductase or the Calvin–Benson–Bassham cycle). The higher fraction of oxidised P_700_ reveals a limitation of the electron flow “uphill” in PSI, confirming that the site of inhibition of the photosynthetic electron‐transfer chain takes place between PSII and PSI.

### Effect of HHQ on the activity of cytochrome *b*_6_ *f*

To probe the exact target of HHQ in the photosynthetic apparatus of *P. tricornutum,* three complementary approaches were used: fast fluorescence transients, *c*‐type cytochrome redox state and electrochromic shift (ECS) measurements. Together, these techniques allowed a detailed understanding of how HHQ affected the photosynthetic electron‐transport chain of diatoms.

**Figure 5:**
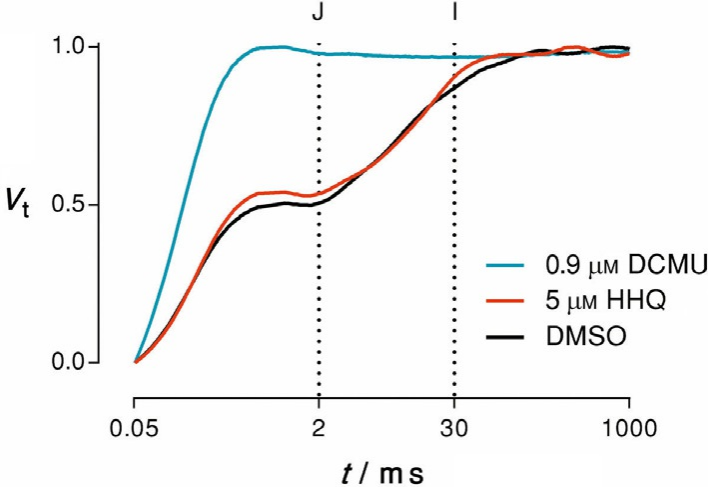
Fast fluorescence transients of *P. tricornutum* after treatment with DMSO (negative control, black), 0.9 μm DCMU (positive control, blue) and 5 μm HHQ (red). The J and I steps of the curve at 2 and 30 ms are indicated with a dotted line. Data are plotted on a logarithmic *x* axis.

Fast fluorescence transients are finely time‐resolved measurements of chlorophyll fluorescence during a saturating multi‐turnover pulse that provide specific information about processes in PSII, but also beyond.^15^ Information is derived from stepwise transitions in fluorescence levels, referred to as the J and I steps at 2 and 30 ms, respectively, and the P step, which describes the time point when maximum fluorescence (*F*_m_) is reached (Figure 5). Whereas DMSO‐treated control cells showed typical shoulders at the J and I steps of the fluorescence transient, DCMU‐treated cells reached *F*_m_ at the J step, which was a typical indicator for PSII inhibition (Figure 5). However, treatment with 5 μm HHQ, the half‐inhibitory concentration of HHQ, only induced a small increase at the J step (2 ms) of the fluorescence transient. This indicated that the primary target of HHQ was not PSII, and thus, led to the hypothesis that the target of HHQ was likely to be downhill of the plastoquinone pool.^15^, ^16^

In parallel, we analysed the ECS of photosynthetic pigments,^17^ which was the change in the absorption spectra of some photosynthetic pigments due to the electric field generated across the thylakoid by the photosynthetic process. The ECS, which can be seen as an in vivo voltmeter, is a powerful and widespread technique to investigate photosynthetic physiology. In *P. tricornutum*, similar to that in other diatoms and stramenopiles,^18^ the ECS is the sum of a linear electric field strength (proportional to the electric field across the thylakoid) and a quadratic component (proportional to the square of the electric field strength). The kinetics of the linear ECS following a saturating laser flash can be used to evaluate the activity of each photosynthetic complex (PSI, PSII, cytochrome *b*_6_ *f*, ATPase). In theory, these kinetics possess three distinct phases: 1) a fast rise of the electric field, representing charge separation due to PSI and PSII activity (<0.1 ms);^19^ 2) a second rise, corresponding to the turnover of cytochrome *b*_6_ *f* (≈10 ms), which pumps additional protons into the lumen; and 3) a relaxation of the electric field as ATPase consumes protons from the lumen to the stroma and converts ADP into ATP (>10 ms).^19^ We also measured the redox state of *c*‐type cytochromes, comprising cytochrome *f* in cytochrome *b*_6_ *f* and cytochrome *c*_6_, which shuttles electrons between cytochrome *b*_6_ *f* and PSI (see the Experimental Section).

**Figure 6:**
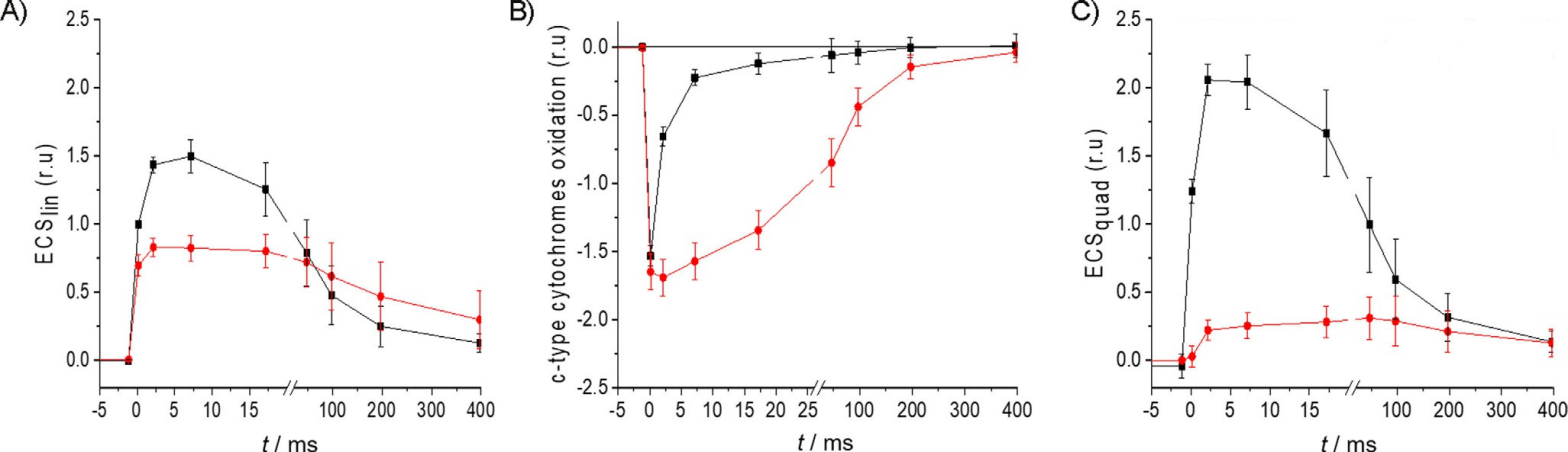
A) Linear ECS, B) *c*‐type cytochrome oxidation states and C) quadratic ECS calculated from absorption changes at *λ*=520, 554 and 564 nm (see the Experimental Section), following a saturating laser flash, given at *t*=0. ▪: control samples (DMSO); •: HHQ treated (50 μm). Error bars represent standard deviation (*n*=5).

As mentioned above, the first phase of ECS kinetics represents charge separation by PSI and PSII immediately after the absorption of a photon. In our measurements, this charge‐separation effect was decreased by 30 % in the presence of HHQ (represented by the first data point after illumination (=0) in Figure 6 A). The charge separations in PSII and PSI lead to electron transfer from water to plastoquinones, and from *c*‐type cytochromes to ferredoxins, respectively. Accordingly, this fast rise of ECS is concomitant with the oxidation of *c*‐type cytochromes by PSI (Figure 6 B), which is similar with and without HHQ, and thus, indicates that PSI photochemistry and electron transfer from cytochromes to P_700_ is unaffected. The 30 % decrease of the ECS fast rise could reflect a slight decrease of PSII activity in the presence of HHQ; however, this cannot explain the almost complete inhibition of photosynthetic activity in the light.

After this fast phase, generating reduced quinols and oxidised *c*‐type cytochromes, the turnover of the cytochrome *b*_6_ *f* complex catalyses the transfer of electrons from the reduced quinols to the oxidised *c*‐type cytochromes. This process is coupled to proton pumping across the thylakoid. The outcome is a phase of reduction of the *c*‐type cytochromes (Figure 6 B) and a second rise in the *trans*‐thylakoid electric field and ECS (Figure 6 A). However, in the presence of HHQ, only a small increase of the electric field in this time frame was observed, and the reduction of *c*‐type cytochromes was 20‐fold slower. This observation identifies the cytochrome *b*_6_ *f* as the main target of HHQ, which explains the overall inhibition of the photosynthetic electron‐transfer rate.

### Suppression of thylakoid proton motive force in darkadapted cells by HHQ

**Figure 7:**
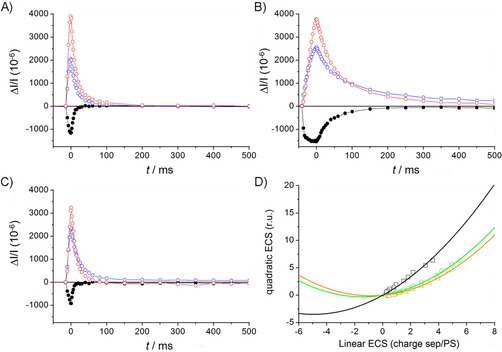
A)–C) Kinetics of linear (blue) and quadratic (red) ECS changes and *c*‐type cytochrome redox state (black) obtained after deconvolution from the kinetics of absorption changes (Δ*I*/*I*) at *λ*=520, 554 and 566 nm, during a 10 ms pulse of saturating red light (before time 0, 4500 μmol photons m^−2^ s^−1^) and the subsequent dark relaxation (after time 0; see the Experimental Section). A) DMSO control, B) treatment with HHQ (50 μm) and C) treatment with membrane potential uncoupler CCCP (15 μm). D) Relationship between quadratic (*y* axis) and linear ECS (*x* axis) in the control (□) and in cells treated with uncoupler (15 μm CCCP, ▵), and with HHQ (50 μm, ○). Data in (D) are obtained from those in (A)–(C). Black squares: control samples (DMSO); red circles: HHQ treated (50 μm). Error bars represent standard deviation (*n*=5).

The last phase of ECS measurements (>10 ms; Figure 6) shows the decay of the electric field and corresponds to the movement of protons from lumen to stroma, as catalysed by ATP synthase. This decay was retarded by HHQ (Figure 6 A). HHQ treatment also decreased the amplitude of the quadratic ECS contribution by about 90 % (i.e., ECS proportional to the square of electric field strength, as shown in Figure 6 C). These two observations hinted at a second effect of HHQ: suppression of the pre‐existing electric field in the dark (Δ*Ψ*_d_), as observed in Bailleul et al.18a To quantify the possible effect of HHQ on the electric field across the thylakoids in dark‐adapted diatoms, we measured the kinetics of the relaxation of the linear and quadratic ECS generated after a saturating pulse of light in untreated *P. tricornutum*, as well as upon treatment with HHQ (50 μm). We also used the membrane potential uncoupler carbonyl cyanide *m*‐chlorophenyl hydrazone (CCCP) as a control to artificially suppress Δ*Ψ*_d_.18a The amplitude of the quadratic versus linear ECS signals were plotted (Figure 7 A–C), giving the same characteristic parabolic function for all treatments, with the vertex indicating the electric field strength in the dark, preceding the light perturbation (expressed in number of charge separations per photosystem; Figure 7 D). The data indicated that a proton motive force (PMF) was maintained across the thylakoid membrane of untreated *P. tricornutum* cells in the dark, with a Δ*Ψ*_d_ corresponding to 5.0±0.6 charge separations per photosystem, similar to previously measured values.18a However, the electric field in the dark was clearly suppressed in the presence of HHQ (Δ*Ψ*_d_=1.4±0.3 charge separations/photosystem), almost as much as with the uncoupler CCCP (Δ*Ψ*_d_=0.8±0.2 charge separations/photosystem).

In the presence of cellular ATP in the plastid in the dark (which comes from mitochondrial respiration activity), plastidic ATPase is able to hydrolyse ATP to ADP, which generates a PMF across the thylakoid membrane.^20^ It is well known in plants, green algae and diatoms that the inhibition of respiratory activity (with uncouplers, mitochondrial inhibitors or under anaerobic conditions) leads to a decrease of the PMF across the thylakoid.^15^, ^20^ Thus, HHQ could suppress the PMF by two means: through an uncoupler effect or through an inhibition of respiration. For that reason, we measured the effect of HHQ on the respiratory activity of *P. tricornutum*.

**Figure 8:**
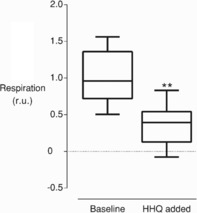
HHQ inhibits respiration in *P. tricornutum*. Oxygen concentration of a diatom culture was tracked over time in a Clark electrode in the dark, and the rate of oxygen consumption was derived from oxygen concentrations 1 min before and after the addition of 50 μm HHQ. Data were normalised to the average untreated respiration rate (r.u.=relative units). Box plots display the median (bar) and 95 % confidence interval. Whiskers indicate maximum and minimum. Asterisks indicate the outcome of the paired *t*‐test (*P*<0.005, *n*=8).

Respiration rates of *P*. *tricornutum* were measured in the dark and derived before and after the addition of HHQ by using a Clark electrode. Respiration rates were more than halved after HHQ addition compared with the respiration rates before the addition of the compound (Figure 8). Accordingly, the observed decrease in electric field strength in the dark was attributed to an inhibition of respiration, rather than through uncoupling of thylakoid charge separation.

## Discussion

A diverse array of interactions between diatoms and bacteria have been documented; however, the physiological mechanisms underlying these interactions are rarely characterised. This study demonstrated the growth inhibitory effects of five different 2‐alkyl‐4‐quinolones on three diatom species, and identified a mode of action for HHQ in *P. tricornutum*. Of all five tested quinolones (Figure 2), HHQ and NHQ had lower IC_50_ concentrations than those of their functionalised homologues (HHQ=1.2–4.9 μm; NHQ=0.16–1.38 μm), which illustrates that in diatoms non‐functionalised quinolones are more potent than that of their functionalised analogues. Although previous studies have shown the toxic effects of HHQ on the coccolithophore *E. huxleyi* 5a and of PHQ on other diatoms,5c, 10 this study builds on previous findings by testing a broader range of quinolones with structural variations. These experiments not only show that diatom growth is inhibited by a wide range of quinolones, but also that some appear to be considerably more potent than that of PHQ. Interestingly, alkylquinolones were more active than that of their corresponding *N*‐oxide derivatives by approximately an order of magnitude, whereas in bacteria–bacteria interactions the *N*‐oxides were much more potent than that of the corresponding non‐functionalised quinolones.^21^

The effects of HHQ were characterised exhaustively by using a wide variety of spectroscopic techniques. These experiments indicate that HHQ inhibits both cytochrome *b*_6_ *f* and, to a lesser extent, PSII in plastids, and oxygen consumption and ATP production in mitochondria. Under actinic light, *Y*(II) values (Figure S2) decreased and the transient reduction of P_700_ was absent (Figure 3); this provides evidence that electron transport in thylakoids is inhibited. This phenotype was clearly visible with 5 μm treatments of HHQ, which was also the IC_50_ value derived from growth experiments and suggested that the inhibition of photosynthesis was the chief mode of growth inhibition. These results were confirmed at all light irradiances under steady‐state illumination (Figure 4), showing that the quantum yield and relative electron‐transport rates of PSI and PSII were impaired by HHQ. The observed inhibition of PSI activity was due to a higher oxidation of P_700_ and not acceptor‐side limitations, which indicated that electron transfer was hampered between PSI and PSII.

Subsequently, the binding site of HHQ was identified as the cytochrome *b*_6_ *f* complex, the activity of which was slowed down 20‐fold (Figure 6). These experiments also showed that charge separation due to the activity of PSII was slightly decreased. These observations are supported by the fluorescence transients (shown in Figure 5), which show a slightly higher amplitude of the O–J phase, indicating an inhibition of electron transfer towards the plastoquinone pool. These data give conclusive evidence that HHQ hinders photosynthesis primarily through the inhibition of cytochrome *b*_6_ *f* and, to a lesser extent, PSII (most likely at the Q_B_ site). Such a result is reinforced by structural similarities between HHQ and plastoquinone/plastoquinol (the mobile electron carrier between PSII and cytochrome *b*_6_ *f*), along with other structurally related molecules with similar inhibitory effects, such as stigmatellin and aurachines.^22^ HHQ also inhibited mitochondrial respiration, as demonstrated by oxygen consumption experiments (Figure 8). The inhibition of respiration leads to a supplementary phenotype. That is, ATP produced by mitochondrial respiration in the dark can be hydrolysed by the chloroplastic ATPase, working “in reverse” and pumping protons into the lumen. Because of this, diatoms, similar to other photosynthetic organisms, generate a PMF across the thylakoid in the dark.18a, 20 Here, the inhibition of mitochondrial respiration by HHQ, and, in turn, the dark PMF (Figure 7), can be visualised by the slower ATPase activity and lower quadratic ECS following a saturating laser flash (Figure 6 C). Complete inhibition of respiration was not achieved, nor was a specific site of inhibition identified. It is nevertheless plausible that HHQ inhibits respiration by hindering electron transport at complex I or III, between which ubiquinone shuttles electrons, similar to the PSII–plastoquinone–cytochrome *b*_6_ *f* system in plastids. Indeed, quinolones have been identified as inhibitors of these complexes in other organisms.11b, 23 Whereas previous studies have shown the effect of 2‐alkyl‐4‐quinolones on respiration in prokaryotes and non‐photosynthetic eukaryotes, this study provides evidence of their inhibition in photosynthetic eukaryotes, and shows that these compounds can simultaneously hinder the function of both photosynthesis and respiration.

Previous studies, mostly on vascular plants, have demonstrated the inhibition of photosynthesis by NQNO, which is known to bind to cytochrome *b*_6_ *f*.^24^ In comparison, much less is known regarding other quinolones. This work suggests that HHQ is a more potent inhibitor of photosynthesis in diatoms than that of NQNO and may be a more favourable molecule for future diatom photo‐physiology studies. For example, a screening of quinolones by Reil et al. with spinach thylakoids showed that non‐functionalised alkylquinolones (e.g., HHQ and NHQ) had only very weak effects on PSII and cytochrome *b*_6_ *f* activity.11c In contrast, this study demonstrates that HHQ strongly inhibits oxygen evolution (Figures S5 and S6), mainly due to the inhibition of cytochrome *b*_6_ *f*. However, it should be noted that many previous studies utilised thylakoid preparations to test quinolones. This presents a problem when comparing them to experiments on whole cells, in which diffusion across membranes and accumulation within cell compartments may influence the effect of quinolones. This is particularly important when considering the unique plastid, thylakoid and cell wall architectures of diatoms.^25^ Nevertheless, physiological differences between diatoms and other photosynthetic organisms appear to define the activity of 2‐alkyl‐4‐quinolones. Such a hypothesis is supported by fluorescence transients from two other microalgae treated with HHQ (Figure S4). In the coccolithophore *E. huxleyi*, for example, treatment with 25 μm HHQ led to an increase of the fluorescence transient at the J and I steps, which could indicate inhibition at PSII and the cytochrome *b*_6_ *f* complex. In contrast, HHQ treatment of the green alga *Dunaliella tertiolecta* only induced an increase of the fluorescence transient at the J step, which suggested that the effect was primarily related to PSII. Indeed, the effect in *D. tertiolecta* was nearly identical to the effect induced by non‐saturating treatments of DCMU. Taken together, these results illustrate how different photosynthetic organisms respond to HHQ in diverse manners, and suggest that the potent effect of HHQ on *E. huxleyi* observed by Harvey et al. was due to the inhibition of photosynthesis.5a

## Conclusion

**Figure 9:**
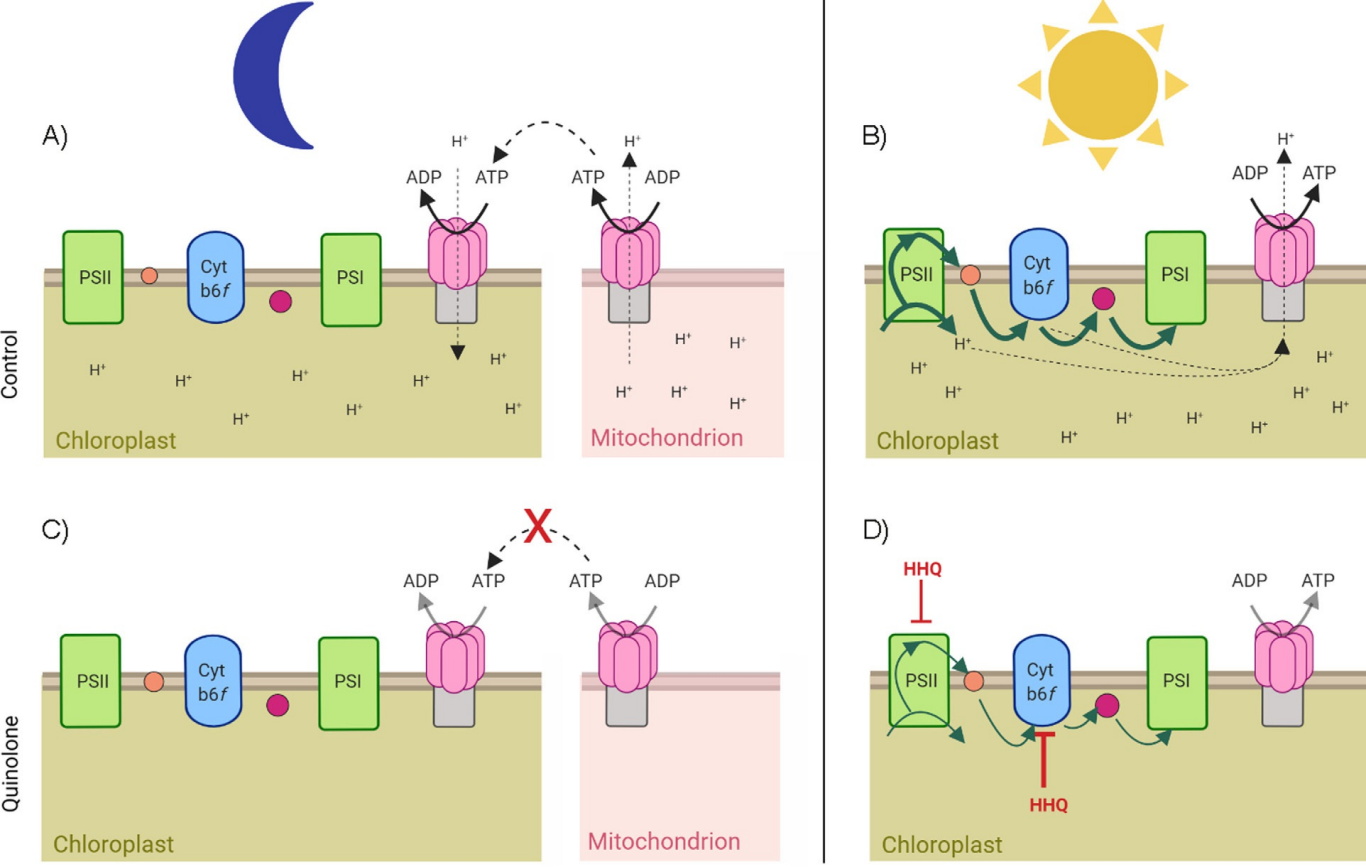
Schematic overview of the electron‐transport chain in chloroplasts and ATPase activity in mitochondria, and their respective functions in dark‐ (A, C) and light‐adapted (B, D) cells. The proposed blockage sites of HHQ are indicated in red (in C and D). Orange circles: plastoquinone; purple circles: cytochrome *c*_6_; Cyt *b*6*f*: cytochrome *b*_6_ *f* complex. Green arrows indicate electron transport and dotted arrows highlight transport of either protons or ATP. The repression of ATP/ADP transformations is indicated with grey arrows. Made in ©BioRender (https://biorender.com).

This study adds detailed physiological data underlying the strong growth inhibitory effect of 2‐alkyl‐4‐quinolones on diatoms, expanding the diverse repertoire of their bioactivity. Furthermore, this study builds on the increasing evidence that 2‐alkylquinolones from bacteria have major roles beyond that of QS as important mediators of interspecies and even interkingdom interactions. This work parallels those on AHLs, which have also been shown to mediate interkingdom interactions in marine environments. For example, AHLs mediate the settling of zoospores in the green macroalga *Ulva*,^26^ whereas tetramic acids, spontaneously generated from certain AHLs, have also been shown to impair photosynthesis in diatoms.^27^ All tested quinolones inhibited photosynthesis, while detailed physiological experiments identified cytochrome *b*_6_ *f* as the chief binding site of HHQ, along with the less severe inhibition of PSII (Figure 9). With the isolation of quinolone‐producing bacteria from marine sources in prior studies,5a, 5c, 7, 8 this study highlights how HHQ could modulate respiratory and photosynthetic activity (Figure 9), and subsequently the proliferation of diatoms in marine environments.

## Experimental Section

**Diatom strains and culture conditions**: *P. tricornutum* (“wild‐type 8”, NEPCC 640) was obtained from the Canadian Centre for the Culture of Microorganisms (CCCM, http://cccm.botany.ubc.ca). Another strain of *P. tricornutum* (“wild‐type 1” Pt 1.8.6) was used for the identification of the site of inhibition of HHQ because the deconvolution of the ECS and *c*‐type cytochromes signals (see below) were previously made on this strain, and we could not rule out that this deconvolution procedure would be as correct in wild‐type 8. *A. minutissimum* (Kützing) Czarnecki was isolated from epilithic biofilms of Lake Constance, Germany. *C. closterium* strain WS3_7 (DCG 0623) was obtained from the Belgian Coordinated Collections of Microorganisms (BCCM, http://bccm.belspo.be). Prior to the experiments, *P. tricornutum* and *C. closterium* cultures were made axenic by treating them with an antibiotic mix (500 μg mL^−1^ penicillin, 500 μg mL^−1^ ampicillin, 100 μg mL^−1^ streptomycin and 50 μg mL^−1^ gentamicin) for a week and replacing the antibiotic‐supplemented medium every second day. *A. minutissimum* was axenified according to a procedure reported by Windler et al.,^28^ and was cultured in a modified bacillariophycean medium,^29^ which instead of soil extract contained F/2 multivitamins, trace metal and silicon/selenium nutrients. *P. tricornutum* and *C. closterium* were cultured in Artificial Seawater Medium (ASW; 34.5 g L^−1^ Tropic Marin, 0.08 g L^−1^ NaHCO_3_) supplemented with Guillard's F/2 (Sigma–Aldrich).^30^ Due to different culture facilities, diatoms were incubated at 18 °C in a 12:12 h light/dark regime at 25 μmol_photons_ m^−2^ s^−1^ for growth experiments, and at 20 °C, with a 16:8 h light/dark regime with a light intensity of 70 μmol_photons_ m^−2^ s^−1^ for PAM fluorometry and Clark electrode experiments. For JTS‐10 experiments, *P. tricornutum* was incubated at 20 °C with a 12:12 h light/dark regime with a light intensity of 70 μmol_photons_ m^−2^ s^−1^.

Cell counts were conducted by using a Multisizer 4e Coulter Counter (Beckmann Coulter). Samples collected for chlorophyll content determination (3 mL of culture) were centrifuged (4500 *g*, 5 min) and the pellet was extracted with methanol (100 μL) and vortexed, followed by acetone (900 μL). The resulting suspension was vortexed and centrifuged once more (18 000 *g*, 2 min). The chlorophyll content of the resulting supernatant was determined according to a procedure by Jeffrey and Humphrey,^31^ in quartz cuvettes on an Ultrospec 2100 pro UV/Vis spectrophotometer (Biochrom).

**Preparation of quinolone solutions**: The 2‐alkyl‐4‐quinolones (HHQ and NHQ) and 2‐alkyl‐4‐quinolone‐*N*‐oxides (HQNO and NQNO) were synthesised as described previously.^21^ Briefly, corresponding 3‐oxoalkanoic acid methyl esters generated from acyl chlorides with Meldrum's acid were used in condensation reactions with aniline to lead to methyl‐3‐phenylamino‐2‐enoates that were subsequently subjected to Conrad‐Limpach cyclisation to give the 2‐alkyl‐4‐quinolones. For the preparation of HQNO and NQNO, HHQ and NHQ were converted into their hydroxyquinoline tautomers as ethyl carbonates that were used in the subsequent *N*‐oxidations.^32^ The resulting ethyl carbonate *N*‐oxides were deprotected to yield the corresponding HQNO and NQNO. PQS was prepared according to a procedure reported by Hradil et al. by using anthranilic acid that was converted into 2‐oxononyl 2′‐aminobenzoate and consequently cyclised in *N*‐methyl‐2‐pyrrolidone (NMP) at 250 °C to PQS.^33^

HHQ, NHQ, PQS, HQNO and NQNO were prepared in DMSO (Sigma–Aldrich) such that the final volume of DMSO added to the cultures or samples was always 0.5 % (*v*/*v*).

**Growth assays**: At the start of the experiment, axenic cultures of *A. minutissimum* and *P. tricornutum* were adjusted to an appropriate cell density by using a Multisizer Coulter Counter. In the case of *C. closterium*, a fixed minimum fluorescence was used with a PAM fluorometer (Walz, Germany), due to the cells rapid sinking rate. Growth assays were conducted in 48‐well plates (Greiner Cellstar, Sigma–Aldrich) and tracked for 6 days following compound addition. Growth was measured by recording chlorophyll autofluorescence, which was normalised to a blank well (filled with ASW), on a Cytation 5 Cell Imaging Multi‐Mode Reader (*λ*_ex_=425 nm/*λ*_em_=685 nm, Biotek). The treatments were randomised among well positions, with three replicates per treatment. The entire growth assay was repeated once more with similar trends. Growth rates were calculated as the logarithmic ratio of cell densities divided by the time interval. The exponential growth phase was thus identified, from which IC_50_ values were derived by using the online AAT Bioquest tool.^34^

**Dual‐PAM experiments**: Dual‐PAM experiments were performed by using a Walz Dual‐PAM 100 fluorometer in dual‐channel mode, equipped with a Dual‐E and a Dual‐DB detector and a cuvette holder with stirrer. Cultures of *P. tricornutum* in the exponential phase were concentrated to a chlorophyll *a* concentration of 40 μg mL^−1^, supplemented with sodium bicarbonate to prevent carbon limitation (16 mm), and adjusted to pH 8.0. For each test, this prepared suspension (2 mL) was treated with a quinolone stock solution or DMSO control (for a final DMSO concentration of 0.5 %, *v*/*v*) and incubated in very low light (resting in the cuvette holder, equivalent to no higher than 10 μmol photons m^−2^ s^−1^ at the surface) for 2 min with stirring, after which the holder was closed. After 10 s in the dark, each sample was exposed to one saturating multi‐turnover pulse (intensity 8000 μmol photons m^−2^ s^−1^, width 800 ms), then low actinic red light was switched on (68 μmol photons m^−2^ s^−1^) followed by a saturating pulse after 30 s. Stirring was switched off immediately before each pulse and restarted immediately afterwards. PSII fluorescence and P_700_ absorbance (*λ*=875 nm minus that at *λ*=830 nm) were recorded in such a manner for each quinolone for each concentration in at least two biological replicates. The initial P_700_ absorbance values were set to equal zero. The PSII quantum yield was also calculated from each of these measurements, by using the same method as that used in JTS fluorescence experiments (see below).

**JTS‐10 experiments**: Photosynthetic parameters of *P. tricornutum* were measured with a JTS (JTS‐10, Biologic, Grenoble, France) equipped with a white probing light‐emitting diode (LED; Luxeon; Lumileds) and a set of interference filters (3–8 nm bandwidth) and cut‐off filters. The device combines absorbance and fluorescence spectroscopy measurements, allowing the activities of PSI and PSII to be studied under the exact same conditions and on the same sample. The actinic light was provided by a crown of red LEDs (*λ*=639 nm, intensities used in this study: 56, 135, 340, 800, and 1500 μmol photons m^−2^ s^−1^). For photosynthesis measurements, cultures of *P. tricornutum* in exponential growth phase were concentrated tenfold by centrifugation (4500 rpm, 4 min) and resuspended in its own supernatant to reach a final concentration in the range of 5–10×10^6^ cells mL^−1^. The centrifuged samples were then left for about 30 min under low light to allow the cells to recover from centrifugation.

**Fluorescence spectroscopy**: In fluorescence spectroscopy mode, the JTS‐10 was equipped with a white probing LED (Luxeon; Lumileds) and a blue filter for detecting pulses. PSII parameters were calculated as reported by Genty et al.^35^ In brief, the maximum quantum yield of PSII and quantum yields in light‐adapted samples were calculated as *F*_v_/*F*_m_=(*F*_m_−*F*_0_)/*F*_m_ and *Y*(II)=(*F*_m_′−*F*)/*F*_m_′′, respectively, in which *F*_0_ is the fluorescence of the dark‐adapted sample, *F*_m_ is the fluorescence when a saturating pulse is applied on a dark‐adapted sample, *F* is the fluorescence of the sample adapted to the actinic light and *F*_m_′′ is the fluorescence when a saturating pulse is applied on light‐adapted sample. The relative electron‐transport rate through PSII (rETR_PSII_) was calculated as rETR_PSII_=*Y*(II)×_I_ *I*, in which *I* is the actinic light irradiance, and then values were all normalised to the value at 1500 μmol photons m^−2^ s^−1^.

**Absorption spectroscopy**: ***P***_**700**_ **measurements**: The redox state of the PSI primary donor (*P*_700_) was calculated as the difference between the kinetic absorption changes at *λ*=705 and 735 nm, to eliminate spectrally flat contributions due to diffusion. Quantum yields of PSI were calculated from the measurements of the absorption changes between *λ*=700 and 735 nm, in the dark (*P*_0_), in the light‐adapted condition (*P*_stat_) and after a saturating pulse (*P*_sp_). *P*_max_, corresponding to 100 % oxidised *P*_700_, was measured as the light minus dark absorption difference in the presence of the PSII inhibitor DCMU (10 mm in ethanol, final concentration of 10 μm). Once normalised to *P*_max_, this allowed the percentage of oxidised *P*_700_ to be calculated in each light condition.

The quantum yields of PSI (*Y*(I)) and the donor‐ (*Y*(ND)) and acceptor‐side (*Y*(NA)) limitations were calculated according to a procedure reported by Klughammer and Schreiber,13a as *Y*(I)=(*P*_sp_−*P*_stat_)/(*P*_max_−*P*_0_), *Y*(ND)=(*P*_stat_−*P*_0_)/(*P*_max_−*P*_0_) and *Y*(NA)=(*P*_max_−*P*_sp_)/(*P*_max_−*P*_0_). The relative electron transport rate through PSI (rETR_PSI_) was calculated as rETR_PSI_=*Y*(I)×*I*, in which *I* is the actinic light irradiance and then values were all normalised to the value at 1500 μmol photons m^−2^ s^−1^.

**ECS and** ***c*** **‐type cytochromes measurements**: Based on previous *P*. *tricornutum* ECS spectra,18a we measured the absorption changes (Δ*I*/*I*; the relative difference of intensity between sample and reference photodiodes) at three wavelengths (*λ*=520, 554, 566 nm). To separate ECS and *c*‐type cytochromes (cytochrome *f* and cytochrome *c*_6_) contributions, and to eliminate flat contributions due to diffusion, we used the following equations: cytochrome *c*=[554]−0.4×[520]−0.4×[566], ECS_lin_=[520]−0.25×cytochrome *c*, and ECS_quad_=[554]+0.15×cytochrome *c*.

To follow the kinetics of those signals following a saturating laser flash, we used a laser dye (LDS 698) pumped by a frequency‐doubled Nd‐YAG laser (Quantel). Before the saturating laser flash was applied, cells were dark adapted for 1 min. For a better comparison, all data in Figure 6 were normalised to the linear ECS value measured immediately after the flash in the control. The photochemical event (“a phase” of ECS kinetics) finished well before 100 μs^19^ and the first experimental point was measured 150 μs after the laser flash.

The dark‐adapted electric field (Δ*Ψ*_d_) measurements were attained from the dark relaxation of the linear and quadratic ECS after a 10 ms pulse of saturating red light (4500 μmol photons m^−2^ s^−1^). ECS data were normalised to the increase of the linear ECS generated after a saturating laser flash (that is, one charge separation per photosystem). We plotted the amplitude of the quadratic versus linear ECS signals during the relaxation of a light‐induced PMF and obtained the parabolic function, which allowed the calculation of the dark electric field, Δ*Ψ*_d_. This experiment was then conducted with cells treated with CCCP (10 mm in ethanol, final concentration 15 μm) and HHQ (10 mm in DMSO, final concentration 50 μm)

**Fast fluorescence transients**: To obtain fast fluorescence transients, an axenic *P. tricornutum* culture was adjusted to 2×10^6^ cells mL^−1^ and supplemented with 40 μm sodium bicarbonate. Quinolone stocks were added to 1 mL of diatom culture in 1.5 mL cuvettes to a final volume of 0.5 % (*v*/*v*). After compound addition, the treated cultures were incubated for 2 min in very low light (resting in the cuvette holder). Finally, the fast fluorescence transients of the cultures were measured with an Aqua Pen instrument (AP‐C 100, Photon Systems Instruments, Drasov, Czech Republic) during a saturating multi‐turnover blue light flash. Fast fluorescence transients were normalised according to a procedure reported by Strasser et al.:^36^ *V*_t_=(*F*−*F*_0_)/(*F*_m_−*F*_0_), in which *V*_t_ is the fluorescence at time *t*, *F*_0_ is the initial fluorescence and *F*_m_ is the maximum fluorescence reached.

**Oxygen electrode measurements**: Oxygen measurements were performed by using a Clark‐type electrode (Hansatech Instruments Ltd.) at 20 °C, with stirring. DMSO and quinolone stocks were added such that the final DMSO concentration never exceeded 0.5 % (*v*/*v*). All experiments began with a 2 min period of complete darkness, followed by addition of HHQ. In the case of photosynthesis measurements, this was then followed by red‐light illumination of 200 μmol photons m^−2^ s^−1^ for the remainder of the test. Measurements were normalised based on the oxygen evolution observed in the dark period. Respiration rate (=oxygen consumption) of intact diatom cells was measured in the dark by calculating the rate of oxygen consumption over time (in 60 s), before and after adding HHQ (*n*=8). Subsequently, the values were divided by the average value before HHQ addition to obtain a ratio in which the average respiration rate of untreated cells was one.

For oxygen measurements on intact diatom cells, exponentially growing *P. tricornutum* (wild‐type 8) cultures were concentrated by centrifugation (4500 *g*, 5 min) to a final concentration of 5 μg chlorophyll *a* mL^−1^ and supplemented with 16 mm NaHCO_3_. For oxygen measurements with thylakoids, thylakoids at a final chlorophyll *a* concentration of 5 μg mL^−1^ were dissolved in 1 mL of 50 mm tricine pH 7.8, 5 mm MgCl_2_, 5 mm K_2_HPO_4_ and 1 mm ATP. In addition, ADP (0.24 mm) and potassium ferricyanide (1.5 mm) were applied. Thylakoid membranes were isolated from exponentially growing *P. tricornutum* wild‐type 8 cultures, following the procedure outlined by Lepetit et al.,^37^ with the following modifications: cells were broken at 13 000 psi, and thylakoid fragments were pelleted by centrifugation at 4 °C for only 10 min at 30 000 *g* (Sorvall) to harvest only larger thylakoid fragments and to reduce the time thylakoids were exposed to the centrifugation forces.

## Conflict of interest

*The authors declare no conflict of interest*.

## Supporting information

As a service to our authors and readers, this journal provides supporting information supplied by the authors. Such materials are peer reviewed and may be re‐organized for online delivery, but are not copy‐edited or typeset. Technical support issues arising from supporting information (other than missing files) should be addressed to the authors.

Supplementary
