# Supplementary material for: The Multifaceted Inhibitory Effects of an Alkylquinolone on the Diatom Phaeodactylum tricornutum
Source: Chembiochem. 2020 Jan 28;21(8):1206–16. doi: 10.1002/cbic.201900612 (PMC7217009; doi:10.1002/cbic.201900612)
Supplement: Supplementary file 1 — Supplementary [file CBIC-21-1206-s001.pdf]

CHEMBIOCHEM

Supporting Information

The Multifaceted Inhibitory Effects of an Alkylquinolone on the Diatom *Phaeodactylum tricornutum*

Lachlan Dow,*[a] Frederike Stock,*[b] Alexandra Peltekis,*[c] Dávid Szamosvári,[d] Michaela Prothiwa,[d] Adrien Lapointe,[a] Thomas Böttcher,[d] Benjamin Bailleul,[c] Wim Vyverman,*[b] Peter G. Kroth,*[a] and Bernard Lepetit*[a]

cbic_201900612_sm_miscellaneous_information.pdf

Supplementary information

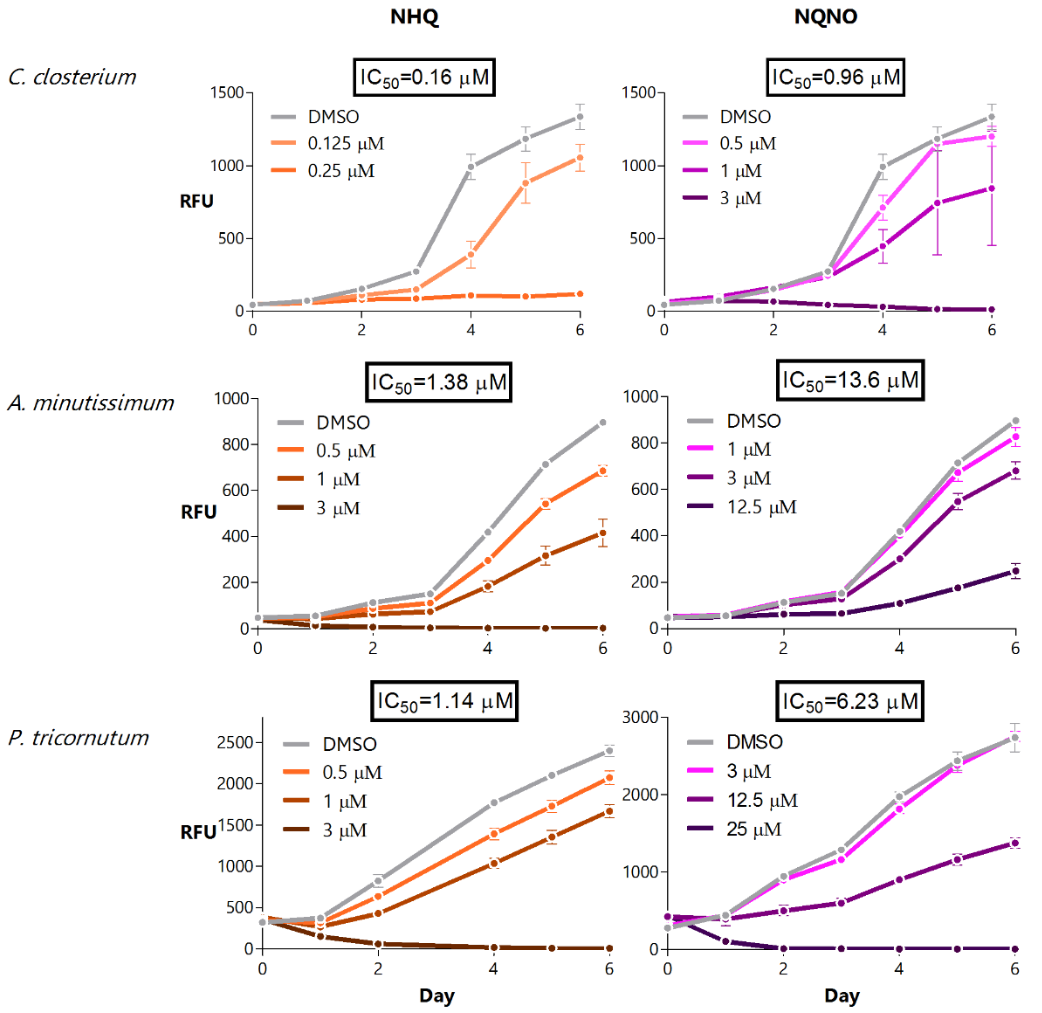

SI 1. Growth of all three diatoms exposed to a range of concentrations of NHQ (left column) and NQNO (right column). The corresponding IC_{50} values are shown in boxes

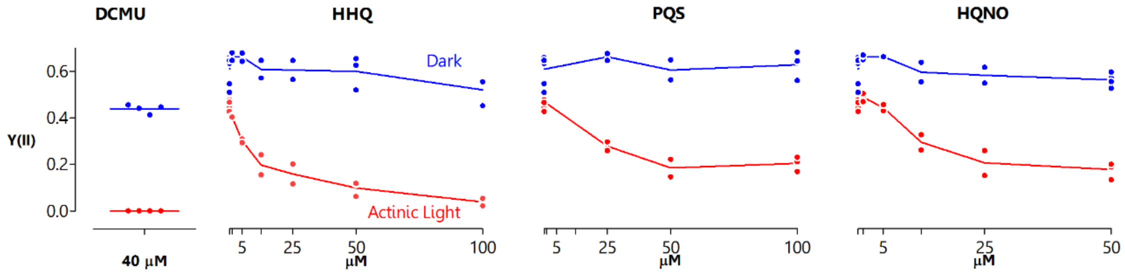

SI 2. PSII quantum efficiency, Y(II), of *P. tricornutum* treated with 40 µM DCMU and concentrations of HHQ, PQS and HQNO measured under dark (blue) and actinic light (red) conditions with a Dual PAM. Y(II) was hardly affected by quinolone treatments under dark conditions while quantum efficiency dropped under actinic light conditions, most severely when HHQ was applied. Each data point is shown with a dot, while the connecting line connects the average of each treatment. Zero µM values are the DMSO control values.

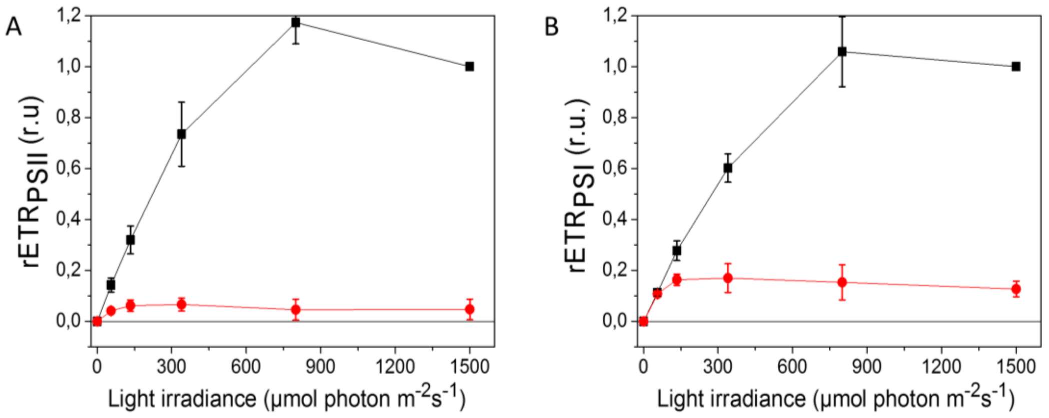

SI 3. Relative electron transfer rate through PSII (A, $rETRP_{PSII}$) and PSI (B, $rETR_{PSI}$) in *P. tricornutum.* control samples (black squares) and samples treated with 50 µM HHQ (red circles). Error bars represent standard deviation (n = 3).

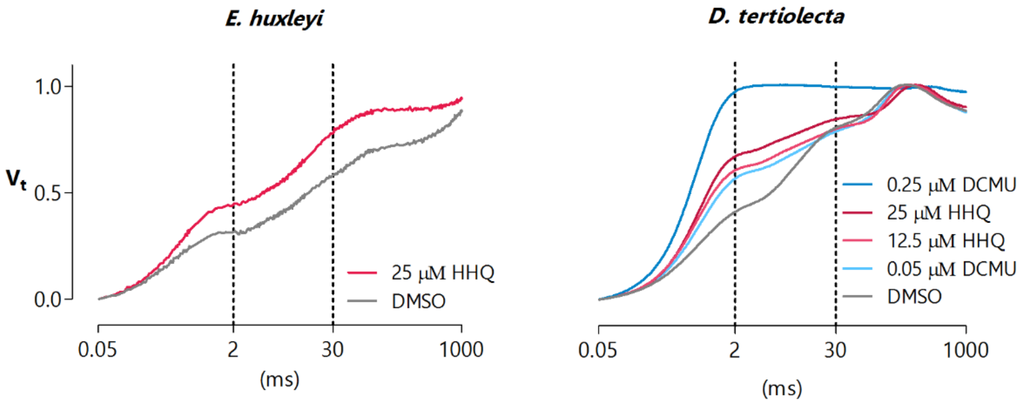

SI 4. Fast fluorescence transients of the coccolithophore *Emiliania huxleyi* and the green alga *Dunaliella tertiolecta,* treated with HHQ (red) or DMSO control (grey). Fluorescence transients of *D. tertiolecta* were also conducted upon treatment with two DCMU concentrations (blue) showing that 0.25 µM DCMU shifts the curve in such a way that F_m is already reached at the J-step of the curve at 2 ms.

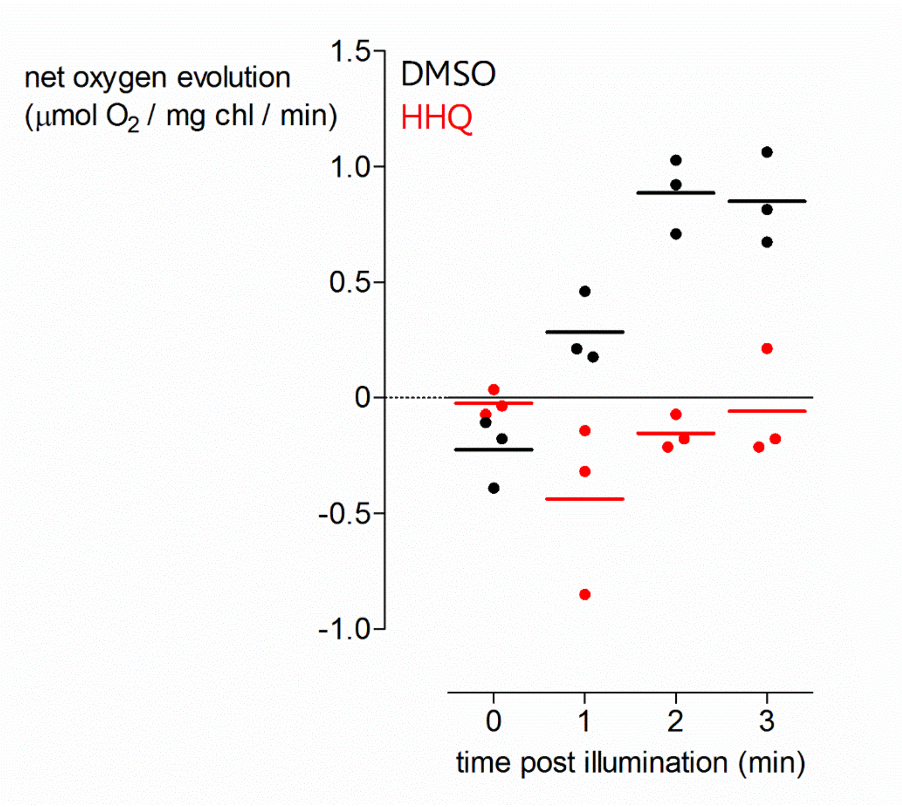

SI 5. Effect of HHQ on Oxygen evolution measurements in *P. tricornutum* cell cultures. Cultures were concentrated to 5 mg chl *a* mL^{-1} and treated with either DMSO (black) or 100 µM HHQ (red). Circles show photosynthetic rate (µmol O_2 mg chlorophyll *a*$^{-1}$ min^{-1}) for each minute post illumination (200 µmol photons m^{-2} s^{-1}) for each replicate, horizontal bars show the mean of the three replicates. Values corresponding to t=0 illustrate the respiration rate (measured as oxygen consumption).

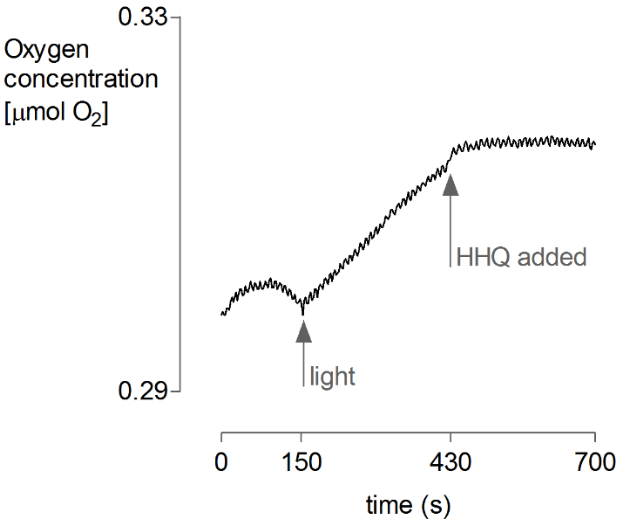

SI 6. Oxygen evolution measurements of thylakoid membrane extracted from *P. tricornutum*. Thylakoid extracts were photosynthetically active during illumination (150 seconds and onwards), however, when treated with 100 µM HHQ (t = 430s), oxygenic photosynthesis halted immediately.
